# HIV prevalence and risk behaviors among female sex workers in Togo in 2017: a cross-sectional national study

**DOI:** 10.1186/s13690-022-00851-0

**Published:** 2022-03-24

**Authors:** Alexandra M. Bitty-Anderson, Fifonsi A. Gbeasor-Komlanvi, Martin Kouame Tchankoni, Arnold Sadio, Mounerou Salou, Patrick A. Coffie, Claver A. Dagnra, Didier K. Ekouevi

**Affiliations:** 1 Centre Africain de Recherches en Epidémiologie et en Santé Publique (CARESP), Lomé, Togo; 2 INSERM U1219, Bordeaux Population Health Research, ISPED, Université de Bordeaux, Bordeaux, France; 3 Programme PACCI – Site ANRS Côte d’Ivoire, CHU de Treichville, Abidjan, Côte d’Ivoire; 4 Département de santé Publique, Faculté des Sciences de la Santé, Université de Lomé, Lomé, Togo; 5 Département des Sciences Fondamentales, Laboratoire de Biologie Moléculaire, Université de Lomé, Lomé, Togo; 6 Département de Dermatologie et d’Infectiologie, Université Félix Houphouët Boigny, UFR des Sciences Médicales, Abidjan, Côte d’Ivoire; 7 CHU de Treichville, Service de Maladies Infectieuses et Tropicales, Abidjan, Côte d’Ivoire; 8 Programme National de Lutte contre le VIH/Sida, les Hépatites virales et les Infections Sexuellement Transmissibles (PNLS/HV/IST), Lomé, Togo

**Keywords:** HIV, Female sex workers, Risk behaviours

## Abstract

**Background:**

The HIV epidemic remains an important public health challenge for the sub-Saharan region. Female Sex Workers (FSW) in this region are among the most vulnerable of the key population groups with HIV prevalence as high as twice that of the general population. The aim of this study was to estimate HIV prevalence and explore sexual risk behaviors among FSW in Togo.

**Methods:**

A cross-sectional study using a Respondent Driven Sampling method was conducted across the six regions of country among FSW in 2017. A comprehensive questionnaire was used to explore socio-demographic characteristics, sexual history, HIV knowledge, and sexual behaviors. HIV rapid tests were used to assess HIV infection.

**Results:**

A total of 1,036 FSW, with a median age of 26 years old [interquartile range (IQR): 22–33], participated in the study, with 49.2% (*n* = 510) of them having reached secondary school. Median age at first sexual intercourse was 20 years old [IQR: 17–25] and estimated number of clients per week was of 5 [IQR: 3–10]. A total of 936 (95.6%) reported the use of a condom during last sexual intercourse with a client and 493 (47.6%) reported the use of a condom during their last sexual intercourse with a partner or husband. HIV prevalence was 13.2% [95% CI: 11.2 – 15.4], and was associated with age (being between 26 and 32 years old; aOR = 4.5; 95% CI: [2.4 – 9.1], *p* < 0.0001) and ≥ 33 years old; aOR = 6.4; 95% CI [3.5 – 12.7], p < 0.0001), education level (being in primary school or less; aOR = 1.7; 95% CI: [1.1–2.6]; *p* = 0.012) and the number of partners per week (more than 2 and 3 partners; aOR = 2.5; 95% CI [1.2—5.2]; *p* = 0.014).

**Conclusions:**

HIV prevalence and sexual risk behaviors remain high among FSW in Togo, despite prevention efforts aimed at curbing this trend. Other factors, such as access and availability of condoms, the social and legal environment in which FSW operate, should be considered for HIV prevention strategies in this population.

**Supplementary Information:**

The online version contains supplementary material available at 10.1186/s13690-022-00851-0.

## Background

The most recent numbers on the HIV epidemic in sub-Saharan Africa (SSA) indicates an overall steady control of the incidence of HIV and greater access to treatment, although the continent is still catching up to the 90–90-90 targets [1, 2]. However, those numbers also show the precariousness of the situation among women, one of the most vulnerable groups in the HIV pandemic, especially in SSA. In this part of the world, in 2019, women and girls accounted for 59% of all new HIV infections compared to 49% in the rest of the world; young women aged 15 to 24 were twice as likely to be living with HIV than men [2, 3]. In addition, SSA adolescent girls (15 to 19 years old) and young women (19 to 24 years old) account for 1 in 4 infections [1].

Another group of populations that are known to be at higher risk of HIV infection are key populations, which include men who have sex with men (MSM), people who inject drugs (PWID), sex workers and prisoners. Although they are a small proportion of the general population, key populations and their sexual partners accounted for more than 60% of new adult HIV infections globally in 2019 [4]. Female Sex Workers (FSW) hence are a key population group with the double burden of being women and belonging to a HIV key population group. Key populations account for 69% of new infections in Western and Central Africa and the risk of acquiring HIV is 30 times higher for sex workers [4]. HIV prevalence among FSW is reported to be 12 times higher than that of the general population. Of new HIV infections in Western and Central Africa, 19% are sex workers, the highest proportion across all regions [2].

The risk of transmission of HIV and other sexually transmitted infections (STI) among FSW is particularly high for several reasons including the behavioral risks for women who engage in sex work with multiple sex partners and report inconsistent use of condoms [5]: condoms available in SSA cover less than half of the need [6]. In addition, FSW, as many other key population groups in SSA face a hostile and challenging social environment, in which access to care is limited due to discrimination, stigmatization and criminalization of sex work [7–9].

Togo is a country of West Africa, with a population of 7.89 million inhabitants in 2018, covering 57,000 square kilometres, an infant mortality of 45.2/ 1,000 and an estimated life expectancy of 61.0 years old [10]. The HIV prevalence in Togo is estimated at 2.3%, with a high prevalence among key population groups estimated between 10 and 15% [11–13]. Among FSW, HIV prevalence was estimated at around 13% in 2011 and 12% in 2015 [14, 15]. Also, data on the treatment cascade among FSW in Togo indicate that 96.8% of FSW know their HIV status and 66.9% are on antiretroviral treatment in 2019 [4]. The use of condoms was estimated to be at 86.4%, lower than other key populations [4]. Strategies put in place by the national government to reduce FSW vulnerabilities to HIV and STI include the use of non-governmental organizations focused on HIV prevention and care, essentially with: 1) a community-based preventive response and a community-based appropriate health care access approach; 2) a synergy between social mobilization of NGOs field teams and health care workers in public and associative HIV care and treatment centers. With this strategy, the following activities have been implemented and remain as main strategies used among FSW in Togo: active screening and treatment of positive cases of STI and HIV, active mobile screening in sex work sites and, bearing of costs associated with pre-therapeutic analyses prior to treatment among FSW diagnosed HIV positive [16].

Going along with the World Health Organization (WHO) recommendations and guidelines on the collection of strategic information, including second generation surveillance for HIV/AIDS, this survey was conducted. Second generation surveillance for HIV/AIDS is “the regular, systematic collection, analysis and interpretation of information for use in tracking and describing changes in the HIV/AIDS epidemic over time” [17]. The aim of our study is to provide an update of epidemiological data on HIV prevalence and risk behaviors among FSW from data derived from a national cross-sectional survey conducted in 2017.

## Methods

### Study design, sampling and recruitment

This study was part of a national bio-behavioral cross-sectional study conducted among key populations (MSM, PWID and FSW) from August to September 2017, in eight cities of Togo. Results from this study only focus on FSW. Participants were recruited using a respondent-driven-sampling (RDS) method. The first seeds were selected based on characteristics such as age and place of work to ensure their representativeness. Each seed was then given three coupons with a unique identification code to recruit other seeds in their network until the required sample size was reached. Prior to the study, a mapping of popular hot spots among FSW (bars, brothels, etc.) was completed with leaders from local FSW-serving non-profit organisations. Inclusion criteria were: i) being 18 years or older, ii) living/working/studying in Togo for a minimum of 3 months at the time of the study, iii) in possession of a recruitment coupon and iv) who had sex in exchange for money as a compensation in the previous 12 months.

### Sample size estimation

The sample size estimation was based on the estimated prevalence of HIV infection among FSW in Togo estimated at 11.7% in 2015 [14]. With a precision of 3% and an assumption of 10% of missing data, the minimum sample size was estimated at 495 participants.

### Data collection

After eligibility screening and written informed consent approval, trained study staff (medical students) administered a structured and standardized questionnaire during a face-to-face interview. The questionnaire was adapted from the Family Health International (FHI) 360 validated guide for bio-behavioral surveys [19] and collected information on socio-demographic characteristics, sexual behaviors and the use of condoms, STIs, HIV testing history, access to health care services, and HIV knowledge.

### Biological analyses

HIV was assessed on site by rapid test (SD Bioline HIV/Syphilis Duo, Abott, Santa Clara, CA, USA with sensitivity for HIV: 99.91%; syphilis: 99.67% and specificity for HIV 99.67% and syphilis 99.72% [18] and each positive result was confirmed with another HIV rapid test, the First Response® HIV 1–2-O Card Test (Premier Medical Corporation Pvt. Ltd., Maharashtra, India; sensitivity: 100% and specificity 99.39% [19]. In case of discordant results, samples were tested with the INNO-LIA® HIV I/II Score (20 T) (Fujirebio, Göteborg, Sweden; sensitivity: 100%; specificity: 100%, [20] line immunoassay.

### Statistical analysis

Descriptive statistics were performed, and results were presented with frequency and proportions for categorical variables. Prevalence rates were estimated with their 95% confidence interval (95% CI). Continuous variables were described with median and interquartile range (IQR). Chi-square or Fisher’s exact tests were used to compare categorical variables. Univariate and multivariate logistic regression were performed with a stepwise-descending selection procedure to identify factors associated with HIV infection. The selection of covariates for multivariate analysis was based on the univariate analyses with factors associated with a *p*-value < 0.20. We deemed a p-value < 0.05 as statically significant for all analyses. Associations in the regression model were expressed as adjusted odds ratio (aOR). All analyses were performed using STATA software (STATA ™ 11.0 College Station, Texas, USA).

### Ethical consideration

This study was approved by the “Comité de Bioéthique pour la Recherche en Santé (CBRS)” (Bioethics Committee for Health Research) from the Togo Ministry of Health. Participants provided written consent prior to participation. Potential participants were told about the study purpose and procedures, potential risks and protections, and compensation. Informed consent was documented with signed consent forms. All FSW’s data were computerized using an identification code and no information that could reveal their identification was entered into the database. In addition, all participants were informed that data collected would be used only for research purposes.

## Results

### Sociodemographic characteristics

A total of 1,036 FSW were recruited for this study. Median age was 26 years old [IQR: 22–33] with almost half of them having reached secondary school (*n* = 510; 49.2%). Most FSW were single (*n* = 839; 81.0%) among which 105 (12.5%) were living with a partner. Fifteen percent (*n* = 163) reported that their partners or husbands had more than one spouse or partner and the median number of children was 1 [IQR: 0–2]. Almost three-fourth of the sample (*n* = 736; 71.0%) indicated that they were the sole support of their family, with an estimated median of two individuals that are dependent on their revenues [IQR: 1 – 4]. For more than half of the sample (*n* = 608; 58.7%), sex work was not their unique stream of income with the majority of those with other activities involved in trade (57.1%; *n* = 347) (Table 1).The median age at first sexual intercourse was estimated to be at 17 years old [IQR: 15–18], while median age at first sexual paid intercourse was estimated to be at 20 years old [IQR: 17–25] (Table 1).

**Table 1:** Sociodemographic characteristics and sexual characteristics among female sex workers in Togo, 2017

Variables	N	n	%
**Age (years),**	1,036		
Median [IQR]		26	[22–33]
**Education**	1,036		
Never been to school		176	17.0
Primary school		297	28.7
Secondary school		510	49.2
College/University		50	4.8
Not specified		3	0.3
**Marital status**	1,036		
Married		144	13.9
Not married/Single		839	81.0
Not specified		53	5.1
**Number of children**	1,036		
Median [IQR]		1	[0–2]
**Are you the sole financial support of your family?**	1,036		
No		278	26.8
Yes		736	71.0
Not specified		22	2.1
**Number of people who depend on your income**	1,036		
Median [IQR]		2	[1–4]
**Age at first sex (years)**	984		
Median [IQR]		17	[15–18]
**Age at first transactional sex (years)**	1,016		
Median [IQR]		20	[17–25]

### HIV risk-related behaviors

Participants reported their median number of clients per week to be 5 [IQR: 3–10] with an estimated price of the most recent sexual act at 5, 000 XOF [IQR: 3000–10000], approximately 7.6 €. Out of the 1,036 participants of this study, 1,019 (98.4%) indicated ever having used a male condom. A total of 986 (95.2%) reported the use of a condom during their last sexual intercourse with a client, and 836 (80.4%) reported the use of a condom systematically (every time) with a client during the preceding 30 days. With husbands or partners, 493 (47.6%) reported the use of a condom during their last sexual intercourse and 348 (33.6%) indicated the systematic use of condom in the preceding 30 days with their husbands or partners. Among those who did not use condoms during their last sexual intercourse with a client (*n* = 42), 29 (69.0%) did not use condoms with their partners either (Table 2).

**Table 2:** Condom use among female sex workers in Togo, 2017 (*N* = 1,036)

Variables	n	%
**Condom use during last sexual intercourse**		
Yes	832	80.3
No	128	12.4
Does not know/does not want to answer	10	1.0
Missing /no response	66	6.4
**Condom use during last sexual intercourse with a client**		
Yes	986	95.1
No	42	4.1
Does not know/does not want to answer	1	0.1
Missing /no response	7	0.7
**Condom use during last 30 days with a client**		
Every time /systematically	836	80.7
Often	137	13.2
Rarely	27	2.6
Never	10	1.0
Does not know/Does not want to answer	1	0.1
Missing	26	2.4
**Condom use during last sexual intercourse with a partner**		
Yes	493	47.6
No	349	33.7
Does not know/does not want to answer	7	0.7
Missing /not applicable (no partner)	187	18.1
**Condom use during last sexual intercourse with a partner**		
Every time /systematically	348	33.6
Often	197	19.0
Rarely	100	9.7
Never	150	14.5
Does not know/Does not want to answer	3	0.3
Missing /not applicable (no partner)	238	23.0

As a measure of condom use consistency, 84.3% (*n* = 831) of FSW having used condoms during the last sexual intercourse with a client also used condoms systematically (every time) with a client during the previous 30 days.

A total of 108 (10.4%) participants reported facing frequently and often condom breakage during sexual intercourse. In terms of knowledge on protective behaviors against HIV, 18.6% (*n* = 191) believed one could not be protected from HIV by using a condom correctly and 33.6% (*n* = 346) believed one could not be protected from HIV by having sexual relations exclusively with a faithful partner. For 205 FSW (19.9%), a person appearing healthy could not be HIV-positive.

### HIV testing and HIV prevalence

The majority of FSW (94.1%; *n* = 895) declared having previously done a HIV test, with 96.6% (*n* = 865) of them knowing their serological status. The most recent HIV test completed was through HIV voluntary counseling and testing (VCT) for the majority (*n* = 440, 49.2%); for 29.8% (*n* = 267) of them during a HIV mass campaign and for 10.8% (*n* = 97), HIV testing was done as part of their routine medical care. Only 42.7% (*n* = 442) reported knowing the serological status of their regular partner. A total of 1,003 FSW (96.8%) agreed to be tested for HIV and prevalence was 13.2% [95% CI: 11.2 – 15.4], with rates ranging from 3.1% in Dapaong in north Togo, 14.8% in the capital city of Lomé in the south and 26.8% in Kara (second city of Togo), in the north of the country as well (*p* = 0.0003).

In multivariate analysis, being aged 26 to 32 (aOR = 4.5; 95% CI: [2.4 – 9.1], *p* < 0.0001), and aged ≥ 33 (aOR = 6.4; 95% CI [3.5 – 12.7], *p* < 0.0001), a level of education of primary school or less (aOR = 1.69; 95% CI: [1.13–2.6]; *p* = 0.0122) and having between 2 and 3, and more partners over the last 7 days (aOR = 2.48; 95% CI [1.2–5.2]; *p* = 0.0140) were associated with HIV infection (Table 3).

**Table 3:** Factors associated with HIV prevalence among female sex workers in Togo in 2017

		**Multivariate Analysis**		
	**n/N**	**Adjusted Odds Ratio**	**95% CI**	***P*-value**
**Age (years)**				** < 0.0001**
< 22	13/325	1		
22–25	16/199	2.03	[0.95–4.44]	0.0685
26–32	44/242	4.54	[2.41–9.09]	< 0,0001
> = 33	59/237	6.39	[3.45–12.66]	< 0,0001
**Educational Level**				**0.0122**
Secondary/University	46/544	1		
Never been to school/Primary school	86/459	1.69	[1.13–2.57]	
**Knowledge of partner’s serological status**				**0.0600**
Yes	40/426	1		
No	92/577	1.49	[0.99–2.29]	
**Number of partners during the last 7 days**				**0.0333**
< 2	15/198	1		
2–3	23/138	2.48	[1.21–5.21]	0.0140
> 3	94/667	1.84	[1.05–3.44]	0.0417

## Discussion

This study examined sexual behaviors of FSW, condom use, HIV prevalence and factors associated with HIV prevalence in this population. As known by previous research and confirmed by this study, FSW are a particularly vulnerable key population group with a prevalence approximately more than four times that of women in the general population, highlighting the ever present burden of the epidemic on this population [21].

When comparing these findings from 2017 to those of 2011 and 2015 in Togo [14, 15], HIV prevalence remained stable among FSW, despite a decrease of the HIV prevalence among women in the general population from 3.1% in 2014 to 2.7% in 2017 [22]. Overall, HIV prevalence remains high among key populations in Togo despite innovative approaches to programs and interventions with non-governmental organizations specializing in care and treatment for key populations. This stabilization could be attributed to several factors, including the fact that HIV programs and interventions could be sparsely suited for this population or that those interventions or programs do not actually reach this population [23]. A large access to antiretroviral treatment and an increase in life expectancy among people living with HIV (PLHIV) could also explain this fact. Prevalence studies should be completed along with pharmacological dosages and viral load testing among HIV-positive FSW in order to better assess the 3X90, a key indicator of HIV care and treatment services. In addition, cohort studies are needed to better document epidemiological data in this population, as well as qualitative studies to gather new insights into this high prevalence in this population.

Just over 10% of FSW admitted to not using condoms on the last sexual intercourse. This proportion is high considering how behavioral interventions for the prevention of HIV among FSW having insisted mainly on the consistent and correct use of condoms [24]. Also, this proportion could be very well underestimated, as research shows actual unprotected sex among FSW to be almost double that reported [5, 25]. Despite behavioral interventions and HIV prevention programs whose main message is the importance of wearing condoms to avoid HIV, the proportion of unprotected sexual acts, especially with clients remains high. This could imply that in the processes influencing condom use, including negotiation and consistent use, structural factors such as economic factors could have more weight in the balance than knowledge and risk perception [26, 27]. A study in South Africa among FSW reported that the main reason for engaging in condomless sex for FSW is to increase earnings, demonstrating the interaction between the economics of sex work and risky sexual behaviors [28]. On the other hand, unsafe sex could also be motivated by clients, who could offer more money for condomless sex, or physical coercion. A study in Vancouver among FSW indicated that accepting more money for sex without a condom was more likely for those who had experience with client violence [29]. This was corroborated by other studies indicating a direct link between violence from clients and inconsistent condom use, hence a higher probability of HIV incidence [30, 31]. Considering the impact of structural determinants, an indirect contributor to the HIV pandemic into HIV prevention programming and policy, among FSW who shoulder an important burden of vulnerabilities, could have an impact into the consistently increasing high incidence of HIV in this population.

In this study, almost two-thirds of FSW reported accidents with their condom use (condom breakage). The use of condoms as a preventive method against HIV implies both the correct and consistent use of condoms. A high prevalence of condom breakage has a direct consequence on the probability of unprotected sexual intercourse, thus of HIV and other STI infections. A qualitative study in Malawi among FSW reported some inadequate behaviors, such as douching, urination or squatting as a mean to prevent HIV, STI and pregnancy after condom breakage. In that study, only 3 out of 18 reported taking appropriate steps after condom breakage (stopping sexual intercourse, seeking medical care including post-exposure prophylaxis for HIV, STI treatment and emergency contraception) [32]. This finding was corroborated by another study in South Africa among FSW [33]. The high occurrence of condom breakage among FSW has been documented in the literature and has consequences on the HIV pandemic. The introduction of the pre-exposure prophylaxis (PrEP) as an intervention among this population could potentially be a key solution toward alleviating the contribution of such incidents to the HIV incidence. Furthermore, additional effort is needed for HIV prevention programs to provide a clear message on the correct use of condoms, and the exact steps needed to be taken in case of condom breakage.

The 90–90-90 strategy aims at diagnosing 90% of people living with HIV, put 90% of them on treatment and for 90% of them to have viral suppression, with the goal of ending the HIV/AIDS epidemic by 2030. In this study, almost all (96.7%) reported knowing their serological status, through VCT and HIV mass campaign. However, less than half of FSW was aware of their partner’s status, although the majority engaged in unprotected sexual intercourse with their partners. A study conducted in Côte d’Ivoire found a similar finding with a low proportion of FSW knowing their partner’ status [34]. Similarly, a study found that unprotected sex acts with partners were 5 to 6 times more frequent than with clients, which was associated with low control in their relationships [35]. Although FSW adopt effective preventive behavior such as getting tested regularly, the effect of this regular testing would be void if they are consistently exposed to risky behaviors from their emotional partners. Knowledge of partners’ status is key to maintaining HIV negative serology. Hence, targeted interventions should include emotional partners as well. In addition, there is the need for additional effort in HIV prevention programs toward partners of FSW with messages on regular HIV testing and condom use with other partners.

Three factors were associated with HIV prevalence: older age, higher number of sexual partners and low level of education. These findings add to the body of evidence on factors associated with HIV infection among FSW in SSA and is similar to trends observed across several HIV seroprevalence studies [15, 36–39]. The correlation of older age and HIV infection could be due to a higher probability of repeated exposure to risk behaviors, with the start of sex work around age 20. Combined with low levels of educational attainment, those risk factors highlight the need for primary prevention interventions among young girls. For example, interventions that would combine a focus on young girls’ empowerment through education (staying in school) as well as HIV prevention and sexual education specifically targeted to them, could both prevent in the long term entry into sex work and vulnerabilities to HIV infection. In addition, a high number of sexual partners was associated with HIV infection, which reiterate the need to explore options such as PrEP in addition to other preventive methods. As of now, PrEP has not been routinely introduced in HIV care and treatment programs and remains largely unavailable as a HIV prevention tool for key populations in Togo.

This study is among the first to report on a high HIV prevalence among FSW in northern Togo, which is a rather unexpected result as the dynamic of the HIV epidemic has found higher prevalence among the general population in the South compared to relatively lower prevalence in the North [40]. This result must be confirmed with further studies. Further quantitative or qualitative studies should explore this aspect of the dynamic of HIV transmission in Togo among key populations in the North and the South of Togo, as compared to the general population. Nevetheless, HIV prevention strategies directed toward key populations should urgently be implemented in that region, especially since studies have also found higher prevalence of Hepatitis B in this region [41].

Some limitations of this study include the nature of the study with the use of a face-to-face questionnaire that could have introduced some bias with the underestimation, underreporting or overestimation of sexual practices and the cross-sectional design of the study from which causality cannot be inferred. Also, other biases such as selection bias could have been introduced with the use of the RDS method, with the convenient sampling of the first seeds and the probable preferential referral behavior of seeds. However, the introduction of these biases were limited with the selection of the first seeds as diverse as possible and the recruitment of FSW in eight cities of Togo to ensure a greater density of social networks.. Despite these limitations, this study was conducted in a large sample and across the different regions of Togo, thus could be generalized to the country and the region. In addition, the use of the RDS method made it possible to recruit a hard-to-reach population, including some participants that may otherwise not been included in the study. Finally, these research findings add to the body of literature on HIV among FSW by demonstrating that despite steps taken for HIV prevention in programming and interventions among FSW, the prevalence has yet to decrease in this population and by confirming that behavioral risk factors remain determinant to HIV infection in this population.

## Conclusions

New research findings from this study highlight the stability of the HIV epidemic among FSW in Togo over the last five years, despite a decrease of the HIV prevalence in the general population, along with a persistence of relatively high levels of risky sexual behaviors. For the last decade until the end of the HIV epidemics, HIV programming for FSW in Togo should emphasize interventions, with a humans’ rights framework (easier or free access to condoms, legal protection from violence), while also integrating clients and partners into HIV interventions. In addition, strengthening awareness campaigns on the consistent and correct use of condoms and offering free HIV screening for clients and partners of FSW could offset the trend in HIV prevalence among FSW. The introduction of PrEP to FSW should also be considered as a tool in preventing new HIV infections. Further research should focus on exploring the impact of structural factors such as violence and access to comprehensive health care services on the vulnerability of FSW to HIV and STI.

## Supplementary Information

**Additional file 1.**

## Data Availability

The datasets used and/or analysed during the current study are available from the corresponding author on reasonable request.

## Abbreviations

AIDS: Acquired Immunodeficiency Syndrome
aOR: Adjusted Odds Ratio
CI: Confidence Interval
FSW: Female Sex Workers
HIV: Human Immunodeficiency Virus
IQR: Interquartile Range
PrEP: Pre-Exposure Prophylaxis
SSA: Sub-Saharan Africa
STI: Sexually Transmitted Infection
VCT: Voluntary Counseling and Testing
XOF: West African CFA Franc

## References

[1] Joint United Nations Programme on HIV/AIDS (UNAIDS). The Western and Central Africa Catch-up plan: putting HIV treatment on the fast-track by 2018. 2017. Available from: https://www.unaids.org/sites/default/files/media_asset/WCA-catch-up-plan_en.pdf

[2] Joint United Nations Programme on HIV/AIDS (UNAIDS). UNAIDS DATA 2020. Joint United Nations Programme on HIV/AIDS(UNAIDS); 2020. Available from: https://www.unaids.org/sites/default/files/media_asset/2020_aids-data-book_en.pdf 12349391

[3] Joint United Nations Programme on HIV/AIDS (UNAIDS). UNAIDS Fact Sheet - World AIDS day. 2020. Available from: https://www.unaids.org/sites/default/files/media_asset/UNAIDS_FactSheet_en.pdf

[4] Programme Conjoint des Nations Unies sur le VIH/SIDA (ONUSIDA). UNAIDS data 2020. 2020 [cited 2020 Aug 26]. Available from: https://www.unaids.org/en/resources/documents/2020/unaids-data

[5] Giguère K, Béhanzin L, Guédou FA, Leblond FA, Goma-Matsétsé E, Zannou DM (2019). Biological validation of self-reported unprotected sex and comparison of underreporting over two different recall periods among female sex workers in Benin. Open Forum Infect Dis.

[6] Joint United Nations Programme on HIV/AIDS (UNAIDS). Prevention Gap Report. 2016. Available from: https://www.unaids.org/sites/default/files/media_asset/2016-prevention-gap-report_en.pdf

[7] Wanyenze RK, Musinguzi G, Kiguli J, Nuwaha F, Mujisha G, Musinguzi J (2017). “When they know that you are a sex worker, you will be the last person to be treated: Perceptions and experiences of female sex workers in accessing HIV services in Uganda”. BMC Int Health Hum Rights.

[8] Lafort Y, Lessitala F, Candrinho B, Greener L, Greener R, Beksinska M (2016). Barriers to HIV and sexual and reproductive health care for female sex workers in Tete, Mozambique: results from a cross-sectional survey and focus group discussions. BMC Public Health.

[9] Nnko S, Kuringe E, Nyato D, Drake M, Casalini C, Shao A (2019). Determinants of access to HIV testing and counselling services among female sex workers in sub-Saharan Africa: a systematic review. BMC Public Health.

[10] The World Bank. Country Profile: Togo. [cited 2021 May 25]. Available from: https://databank.worldbank.org/views/reports/reportwidget.aspx?Report_Name=CountryProfile&Id=b450fd57&tbar=y&dd=y&inf=n&zm=n&country=TGO

[11] Ekouevi DK, Dagnra CY, Goilibe KB, Tchounga B, Orne-Gliemann J, Salou M (2014). HIV seroprevalence and associated factors among men who have sex with men in Togo. Rev Epidemiol Sante Publique.

[12] Comité Nationale de Lutte contre le SIDA (CNLS) Togo. Enquête de surveillance comportementale et biologique des IST/VIH/SIDA _ ESCOMB 2015. 2015. Available from: https://www.unaids.org/sites/default/files/country/documents/GIN_2017_countryreport.pdf

[13] USAID Project Search: Research to prevention. L’analyse des facteurs de risques liés au VIH et des écueils à l’accès aux services des travailleuses de sexe et des hommes ayant des rapports sexuels avec les hommes au Togo. The Johns Hopkins University; 2014. Available from: https://www.jhsph.edu/research/centers-and-institutes/research-to-prevention/publications/togo-brief-french.pdf

[14] Teclessou JN, Akakpo S, Gbetoglo D, Koumagnanou G, Singo A, Pitché P (2017). HIV prevalence and behavioral studies among female sex workers in Togo in 2015. Bull Soc Pathol Exot 1990.

[15] Pitché P, Gbetoglo K, Saka B, Akakpo S, Landoh DE, d’Alméida S (2013). HIV prevalence and behavioral studies in female sex workers in Togo: a decline in the prevalence between 2005 and 2011. Pan Afr Med J.

[16] Conseil National de Lutte contre le SIDA et les IST. Rapport d’activite sur la riposte au VIH/SIDA au Togo. 2015.

[17] World Health Organization. WHO | Second Generation Surveillance for HIV/AIDS. WHO. [cited 2021 May 25]. Available from: https://www.who.int/hiv/topics/surveillance/2ndgen/en/

[18] Abbott. SD Bioline HIV/SYPHILIS duo: Fiche Technique.

[19] World Health Organization. Product: First Response HIV 1–2–0 Card test. (WHO prequalification of In Vitro diagnostics programme: Public Report).

[20] Innogenetics N.V. INNO-LIA HIV I/II Score.

[21] Programme Conjoint des Nations Unies sur le VIH/SIDA (ONUSIDA). Togo. [cited 2021 Mar 18]. Available from: https://www.unaids.org/en/regionscountries/countries/togo

[22] Programme National de Lutte contre le SIDA et les IST. Rapport Annuel 2017 ds activités du PNLS-IST (Togo). Ministère de la Santé et de la Protection Sociale Togo; 2017. Available from: https://www.pnls.tg/rapports/RAPPORT%20ANNUEL%20PNLS%202017.pdf

[23] HIV prevention among key populations. [cited 2022 Mar 2]. Available from: https://www.unaids.org/en/resources/presscentre/featurestories/2016/november/20161121_keypops

[24] Okafor UO, Crutzen R, Aduak Y, Adebajo S, Van den Borne HW (2017). Behavioural interventions promoting condom use among female sex workers in sub-Saharan Africa: a systematic review. Afr J AIDS Res AJAR.

[25] Lépine A, Treibich C, Ndour CT, Gueye K, Vickerman P (2020). HIV infection risk and condom use among sex workers in Senegal: evidence from the list experiment method. Health Policy Plan.

[26] Namey E, Perry B, Headley J, Yao AK, Ouattara ML, Shighata C (2018). Understanding the financial lives of female sex workers in Abidjan, Côte d’Ivoire: implications for economic strengthening interventions for HIV prevention. AIDS Care.

[27] MohammadiGharehghani MA, Khosravi B, Irandoost SF, Soofizad G, YoosefiLebni J (2020). Barriers to condom use among female sex workers in Tehran, Iran: a qualitative study. Int J Womens Health.

[28] George G, Nene S, Beckett S, Durevall D, Lindskog A, Govender K (2019). Greater risk for more money: the economics of negotiating condom use amongst sex workers in South Africa. AIDS Care.

[29] Deering KN, Lyons T, Feng CX, Nosyk B, Strathdee SA, Montaner JSG (2013). Client demands for unsafe sex: the socioeconomic risk environment for HIV among street and off-street sex workers. J Acquir Immune Defic Syndr 1999.

[30] Deering KN, Bhattacharjee P, Mohan HL, Bradley J, Shannon K, Boily M-C (2013). Violence and HIV risk among female sex workers in Southern India. Sex Transm Dis.

[31] Decker MR, Wirtz AL, Pretorius C, Sherman SG, Sweat MD, Baral SD (2013). Estimating the impact of reducing violence against female sex workers on HIV epidemics in Kenya and Ukraine: a policy modeling exercise. Am J Reprod Immunol N Y N 1989.

[32] Twizelimana D, Muula AS (2020). Actions taken by female sex workers (FSWs) after condom failure in semi urban Blantyre, Malawi. BMC Womens Health.

[33] Mukumbang FC (2017). Actions of female sex workers who experience male condom failure during penetrative sexual encounters with clients in Cape Town: Implications for HIV prevention strategies. South Afr J HIV Med.

[34] Becquet V, Nouaman M, Plazy M, Masumbuko J-M, Anoma C, Kouame S (2020). Sexual health needs of female sex workers in Côte d’Ivoire: a mixed-methods study to prepare the future implementation of pre-exposure prophylaxis (PrEP) for HIV prevention. BMJ Open.

[35] Luchters S, Richter ML, Bosire W, Nelson G, Kingola N, Zhang X-D (2013). The contribution of emotional partners to sexual risk taking and violence among female sex workers in Mombasa, Kenya: a cohort study. PloS One.

[36] Chabata ST, Hensen B, Chiyaka T, Mushati P, Mtetwa S, Hanisch D (2019). Changes over time in HIV prevalence and sexual behaviour among young female sex-workers in 14 Sites in Zimbabwe, 2013–2016. AIDS Behav.

[37] Bowring AL, Ketende S, Billong SC, MfochiveNjindam I, Rao A, Decker MR (2019). Characterizing sociostructural associations with new HIV diagnoses among female sex workers in Cameroon. J Acquir Immune Defic Syndr 1999.

[38] Jonas A, Patel SV, Katuta F, Maher AD, Banda KM, Gerndt K (2020). HIV prevalence, risk factors for infection, and uptake of prevention, testing, and treatment among female sex workers in Namibia. J Epidemiol Glob Health.

[39] Hensen B, Chabata ST, Floyd S, Chiyaka T, Mushati P, Busza J (2019). HIV risk among young women who sell sex by whether they identify as sex workers: analysis of respondent-driven sampling surveys, Zimbabwe, 2017. J Int AIDS Soc.

[40] Sadio AJ, Gbeasor-Komlanvi FA, Konu YR, Sewu EK, Zida-Compaore W, Salou M (2019). Prevalence of HIV infection and hepatitis B and factors associated with them among men who had sex with men in Togo in 2017. Med Sante Trop.

[41] Ekouevi DK, Larrouy L, Gbeasor-Komlanvi FA, Mackiewicz V, Tchankoni MK, Bitty-Anderson AM (2020). Prevalence of hepatitis B among childbearing women and infant born to HBV-positive mothers in Togo. BMC Infect Dis.

